# From social learning mechanisms to cultural participation: the mediating role of learning culture in Miao embroidery communities

**DOI:** 10.3389/fpsyg.2026.1888090

**Published:** 2026-09-25

**Authors:** Rong Xiong, Yaoping Liu

**Affiliations:** 1 Guiyang University, Guiyang, Guizhou, China; 2 Faculty of Liberal Arts, Rajamangala University of Technology Thanyaburi, Pathum Thani, Thailand; 3 Department of Global Buddhism, Institute of Science, Innovation and Culture, Rajamangala University of Technology Krungthep, Bangkok, Thailand

**Keywords:** cultural participation, intangible cultural heritage, learning culture, Miao embroidery, social learning mechanisms, traditional crafts

## Abstract

Research into the sustainability of traditional crafts has focused on preservation, digitalization, design innovation, and creative economy; however, there has been relatively little emphasis on the micro-level dynamics through which community learning leads to sustainable Participation in culture. This study examines the relationships between social learning processes, learning culture, cultural Participation, and cultural outcomes in Miao embroidery communities in Guizhou, China. Social learning is defined by three mechanisms: community interaction, observational learning, and Participation in practice. In a quantitative cross-sectional research design, data were gathered from 280 purposively sampled Miao embroiderers and analyzed using partial least squares structural equation modeling (PLS-SEM). The findings show that community interaction, observational learning, and Participation in practice are positively related to learning culture, which in turn is positively related to cultural Participation. Cultural Participation is the strongest predictor of attachment to cultural identity, including embroidery practice and Participation in cultural events. Further, mediation analyses show that learning culture mediates the relationship between social learning processes and cultural Participation, while cultural Participation mediates the relationship between learning culture and cultural outcomes. The implication is that the sustainability of Miao embroidery depends not only on learning itself but also on the process of institutionalizing learning through a culture of community learning and Participation in culture.

## Introduction

Crafts, as part of intangible cultural heritage, cannot be regarded simply as esthetic products or professional abilities; rather, they should be understood as socially situated knowledge systems involving meaning-making and collective memory, transmitted through everyday social activities and interactions. In other words, the knowledge involved in crafts is usually acquired not through formal education but through observation, social interaction, imitation, correction, and Participation in community practices. Therefore, sustainable traditional crafts are only possible when their social processes of production and maintenance are preserved for future generations. Prior studies have highlighted the significance of place specificity, memories, cultural identity, and socially structured knowledge in crafting practices (Toraldo et al., 2018; Połeć and Murawska, 2021; Guo and Ahn, 2023; Li et al., 2023). In parallel, the processes of globalization, socio-economic change, commodification of cultures, and other broader social transformations have been progressively interfering with the mechanisms through which such socially transmitted socio-cultural knowledge is possible (Zhang and Sharudin, 2024; Du and Jiang, 2024; Tan et al., 2025).

In the case of Miao embroidery in Guizhou, these broader transformations are reflected in concrete challenges to inheritance within local communities. Miao embroidery has long been transmitted through family-based learning, community interaction, observation of experienced embroiderers, and repeated Participation in everyday craft practices. However, rural-to-urban migration, changing livelihood patterns, tourism-oriented commercialization, and the increasing separation between younger generations and traditional community spaces have weakened these informal learning environments. As embroidery becomes increasingly linked to market products, cultural exhibitions, and heritage promotion, the risk is that the craft may remain visible as a cultural symbol. At the same time, the social processes that sustain its knowledge transmission become fragile. Local embroidery communities, therefore, face not only the challenge of preserving motifs and techniques but also the deeper problem of maintaining the everyday learning relationships through which embroidery skills, cultural meanings, and identity attachments are reproduced. This condition makes Miao embroidery an important context for examining how micro-level social learning mechanisms are transformed into a learning culture and sustained cultural Participation.

There is considerable progress made in the area of sustainable use of traditional craftsmanship in the form of heritage protection, digital preservation, design innovation, and creative economy and cultural marketing initiatives (Pagán et al., 2020; Mudau et al., 2024; Chen and Wang, 2024; Kofler and Walder, 2024). In this way, the social and cultural importance of traditional crafts has become more evident than before. Nonetheless, these studies have mostly focused on macro-level strategies and external forms of preservation, leaving the social micro-processes related to craft practices unexplored. In particular, limited empirical attention has been paid to how community interaction, observational learning, and participation in practice function as social learning mechanisms that foster a learning culture and, in turn, sustain cultural participation. The reason this gap is of utmost importance is that cultural Participation cannot be measured by attendance, the number of productions, or marketing efforts; it should be seen as a behavior rooted in culture, identity, and commitment to practice. The lack of such a framework will result in cultural preservation being seen as nothing more than representation, rather than the actual practice itself.

To fill this gap, the current research proposes a conceptual and empirical framework that explains the relationships among social learning, culture, Participation, and the outcomes of cultural Participation in Miao embroidery communities in Guizhou, China. This paper will approach social learning not as a singular higher-level concept, but through three mechanisms: social interaction, observational learning, and Participation. These mechanisms are expected to shape learning culture as a collective structure through which knowledge sharing, informal correction, intergenerational transmission, and community-based learning become institutionalized. Learning culture is then positioned as a mediating mechanism that transforms social learning mechanisms into cultural Participation. At the same time, cultural Participation is examined as a proximal driver of attachment to cultural identity, embroidery engagement, and Participation in cultural events. The research in this article uses PLS structural equation modeling (PLS-SEM) to present a micro-sociological theory explaining how traditional craft sustainability is produced. According to the findings, sustainability is not simply an issue of preservation, digitalization, and innovation, but of establishing learning and active involvement in culture.

## Literature review

Culture is dynamically constituted through interconnected systems of people, places, and practices (Causadias, 2020). The learning process in traditional crafts is viewed as social, physical, and practical rather than as an organized transfer of knowledge. The learning of craft involves the possession of knowledge that is contextualized and embodied, gained through observation, imitation, correction, involvement, and Participation in social practices. Therefore, to sustain craft traditions, it is not only necessary to have cultural knowledge but also to have the right social conditions that enable people to participate actively in their culture. Previous studies have shown that learning crafts needs to involve active engagement in social practice since cultural knowledge, meaning, and technical aspects cannot be taught through verbal transmission alone (Aytekin and Rızvanoğlu, 2019; Partarakis et al., 2020; Suryanti et al., 2022; Li et al., 2023; Guo and Ahn, 2023).

China-based studies on Miao embroidery have emphasized its significance as an ethnic cultural symbol, a carrier of historical memory, and a visible form of intangible cultural heritage. In Guizhou, Miao embroidery is closely associated with women’s craft practice, family-based transmission, community learning, and the symbolic use of motifs, colors, and needlework techniques to express cultural identity and collective memory (Li et al., 2023; Wang and Sun, 2022). Recent studies on the innovative development of Miao embroidery have further shown that digital design, pattern databases, creative product development, cultural tourism, and market revitalization are increasingly used to enhance its visibility, productivity, and economic value (Du and Jiang, 2024; Zhang and Sharudin, 2024). More recent design-oriented research also suggests that digital tools and intelligent pattern generation can support broader Participation in embroidery practice and provide technical pathways for the sustainable design of Miao embroidery as an element of intangible cultural heritage (Zhang et al., 2025). However, these studies remain largely concentrated on cultural representation, design transformation, technological innovation, and economic revitalization. They pay less attention to the micro-social processes through which embroidery knowledge is learned, corrected, practiced, internalized, and transmitted within local communities. This limitation reinforces the need to examine Miao embroidery inheritance through community interaction, observational learning, Participation in practice, learning culture, and cultural Participation.

The three methods by which this happens include community interactions, observational learning, and Participation in practice. The first method is used to enable knowledge circulation through discussions, criticism, advice, problem-solving, and more. The second method provides examples of proper practice and, therefore, helps less skilled artisans learn proper practices. Thirdly, Participation in practice helps actualize and solidify the observed knowledge. The three mechanisms are interdependent because community interactions clarify meanings, observational learning provides models of practice, and practice solidifies knowledge through application. Studies on social learning, craft transmission, and heritage sustainability demonstrate that knowledge transfer is durable only with continuous engagement with culture (Scavarelli et al., 2021; Mudau et al., 2024; Chen and Wang, 2024; Li et al., 2024). Therefore, the effectiveness of these three mechanisms will determine whether knowledge transfer occurs individually or collectively.

However, the use of social learning mechanisms does not necessarily ensure sustainable cultural Participation. These social learning mechanisms need to be institutionalized within a learning culture. A learning culture refers to the set of shared values, routines, norms, and practices through which knowledge exchange, correction, retention, and transmission take place within a community (Malik and Garg, 2017). A learning culture can bring coherence from fragmented learning experiences through its social dimension, which allows knowledge to transcend the boundaries of individual artisans and be transferred to society at large (Al-Kurdi et al., 2018; Ting, 2023; Chen and Wang, 2024; Mudau et al., 2024). As such, cultural Participation becomes an action-oriented aspect of learning culture, encompassing not only Participation in craft-related activities but also cultural values, identity, commitment, and attachment (Li et al., 2023). Unfortunately, existing empirical evidence has not sufficiently examined how community interactions, observational learning, and active Participation translate into cultural Participation through the learning culture. This paper contributes to filling this research gap by considering learning culture as the mediating mechanism between social learning mechanisms and cultural Participation.

### Hypothesis development

#### Social learning mechanisms and learning culture

In traditional craft communities, social learning is not an abstract or uniform process, but is enacted through concrete mechanisms embedded in everyday practice. In this study, social learning is operationalized through three mechanisms: community interaction, observational learning, and Participation in practice. Community interaction promotes the transfer of knowledge through communication, feedback, coaching, and collaboration among artisans on problems. Collaborative interaction and engagement can strengthen learning processes (Qureshi et al., 2023). Observation is another process through which a person gains insight into methods, symbols, standards, and cultural significance by observing experts. Such situated learning resembles cognitive apprenticeship, in which novices develop knowledge through observation, guided practice, and participation (Collins et al., 2018). Practice provides an opportunity to engage in practical action in which the person verifies, corrects, and solidifies their knowledge. Such processes play an important role in the transfer of traditional crafts because the knowledge involved cannot be transmitted solely through instruction (Guo and Ahn, 2023; Chen and Wang, 2024; Li et al., 2023).

Learning cultures arise from the repeated practice of learning processes in a community setting. They involve shared processes, routines, expectations, and norms that facilitate the circulation, correction, preservation, and transmission of knowledge between members and across generations. Interaction in communities improves the culture of learning by fostering an environment where meanings can be negotiated and practical knowledge exchanged. The process of observational learning is important because it establishes standards and models of practice. Participatory practice in a community setting fosters a learning culture by institutionalizing learning as a continuous experience. Previous literature suggests that interaction, collective practice, and knowledge sharing in communities increase the likelihood of having established learning processes (Li et al., 2020; Wang and Sun, 2022; Mudau et al., 2024).

This study operationalizes social learning through community interaction, observational learning, and Participation in practice because these three mechanisms correspond to the main ways tacit craft knowledge is transmitted in Miao embroidery communities. Community interaction reflects the relational exchange of advice, feedback, correction, and cultural meaning. Observational learning reflects modeling and imitation, through which artisans internalize techniques, motifs, standards, and symbolic meanings by watching skilled practitioners. Participation in practice reflects embodied engagement, where knowledge is tested, corrected, and stabilized through repeated embroidery activities. Thus, the three mechanisms are not intended as an exhaustive classification of social learning theory, but as a context-specific operationalization suited to traditional craft inheritance.

Therefore, the following hypotheses are suggested:

*H1*: Community interaction positively affects the learning culture.

*H2*: Observational Learning positively affects the learning culture.

*H3*: Participation in Practice positively affects learning culture.

#### Learning culture and cultural participation

Learning cultures form the social context in which individual learning becomes a part of sustainable Participation in cultural life. When knowledge exchange, social feedback, and learning come to dominate a community, members will not only be provided with craft knowledge but also motivated to participate in the culture as an aspect of communal life. Thus, cultural Participation will go beyond mere attendance and frequent Participation to become the behavioral expression of cultural values, cultural identification, and a desire for cultural sustainability. A sound learning culture helps members understand the meaning and cultural significance of crafts and motivates them to participate in cultural activities. Research has shown that communities oriented toward learning can increase engagement, affirm their cultural identity, and sustain their culture (O’Meara et al., 2019; Li et al., 2023; Mudau et al., 2024). Based on this, the following hypothesis is formulated:

*H4*: Learning culture positively affects cultural participation.

#### Cultural participation and cultural outcomes

Cultural participation functions as the most immediate pathway through which learning culture is associated with cultural outcomes. In craft communities, Participation involves more than just technical Participation; it becomes a process through which the individual adopts cultural symbols, values, identity, and shared memories. Repeated Participation in Miao embroidery can strengthen identity attachment, as participants feel that embroidery is part of their identity and community identity. Participatory action can also enhance involvement in embroidery by strengthening commitment to the craft, developing skills, and maintaining continuity in production. Participation in culture can motivate people to attend public cultural events such as festivals, exhibitions, and ceremonies. Previous research has shown that cultural Participation is closely linked to identity attachment, belonging, engagement, and community participation (Shepherd et al., 2017; Grossi et al., 2019; Kim, 2020; Su, 2020). Therefore, the following hypotheses are proposed:

*H5*: Cultural Participation has a positive effect on cultural identity attachment.

*H6*: Cultural Participation positively affects engagement in embroidery.

*H7*: Cultural Participation has a positive effect on Participation in cultural events.

#### Mediating role of learning culture and cultural participation

The proposed mediation logic is based on the assumption that learning mechanisms in craft communities do not automatically become sustained cultural Participation. Community interaction, observational learning, and Participation in practice provide the conditions for knowledge circulation, modeling, and the acquisition of embodied skills. However, these mechanisms require a stable social structure to become durable. Learning culture serves this function by institutionalizing repeated learning activities into shared norms, routines, expectations, and collective practices. Studies on community-based learning and heritage transmission suggest that learning becomes sustainable when it is embedded in social structures that support knowledge sharing, collective Participation, and continuity across members and generations (Baldridge et al., 2017; Edwards, 2019; Guo and Ahn, 2023; Mudau et al., 2024). In traditional craft communities, this means that interaction, observation, and practice are more likely to generate cultural Participation when they are stabilized as a community learning culture. Therefore, learning culture is expected to mediate the relationships between social learning mechanisms and cultural Participation.

Cultural Participation is also expected to function as a second mediating mechanism linking learning culture to cultural outcomes. Learning culture provides a normative and social environment for knowledge transmission, but cultural outcomes such as identity attachment, practical engagement, and Participation in cultural events require active behavioral involvement. Previous studies have shown that cultural Participation is closely linked to identity formation, belonging, engagement, and community involvement, as it allows individuals to internalize cultural values through repeated practice and shared experience (Grossi et al., 2019; Kim, 2020; Su, 2020; Li et al., 2023). Research on community attachment and Participation further indicates that social structures influence individual outcomes through participation-based mechanisms rather than through normative conditions alone (Kim et al., 2023). In the context of Miao embroidery, learning culture is therefore expected to contribute to cultural identity attachment, engagement in embroidery, and Participation in cultural events. Based on this sequential logic, the following hypotheses are proposed:

*H8*: Learning culture mediates the relationships between social learning mechanisms and cultural Participation.

*H9*: Cultural Participation mediates the relationship between learning culture and cultural outcomes.

### Research model

In this study, a theoretical model has been developed to explain how social learning processes in Miao embroidery groups evolve into patterns of cultural engagement and cultural outcomes. Social learning has been defined by three processes: social interaction, observation, and practice. These mechanisms are expected to shape learning culture as a collective structure that institutionalizes knowledge sharing, informal correction, repeated practice, and intergenerational transmission. Learning culture, in turn, promotes cultural Participation, which represents the behavioral realization of shared cultural knowledge and values. Cultural Participation is expected to strengthen further attachment to cultural identity, engagement in embroidery, and Participation in cultural events. The model also assumes two mediation pathways: learning culture mediates the effects of social learning mechanisms on cultural Participation. In contrast, cultural Participation mediates the relationship between learning culture and cultural outcomes. The proposed research model is presented in Figure 1.

**Figure 1:**
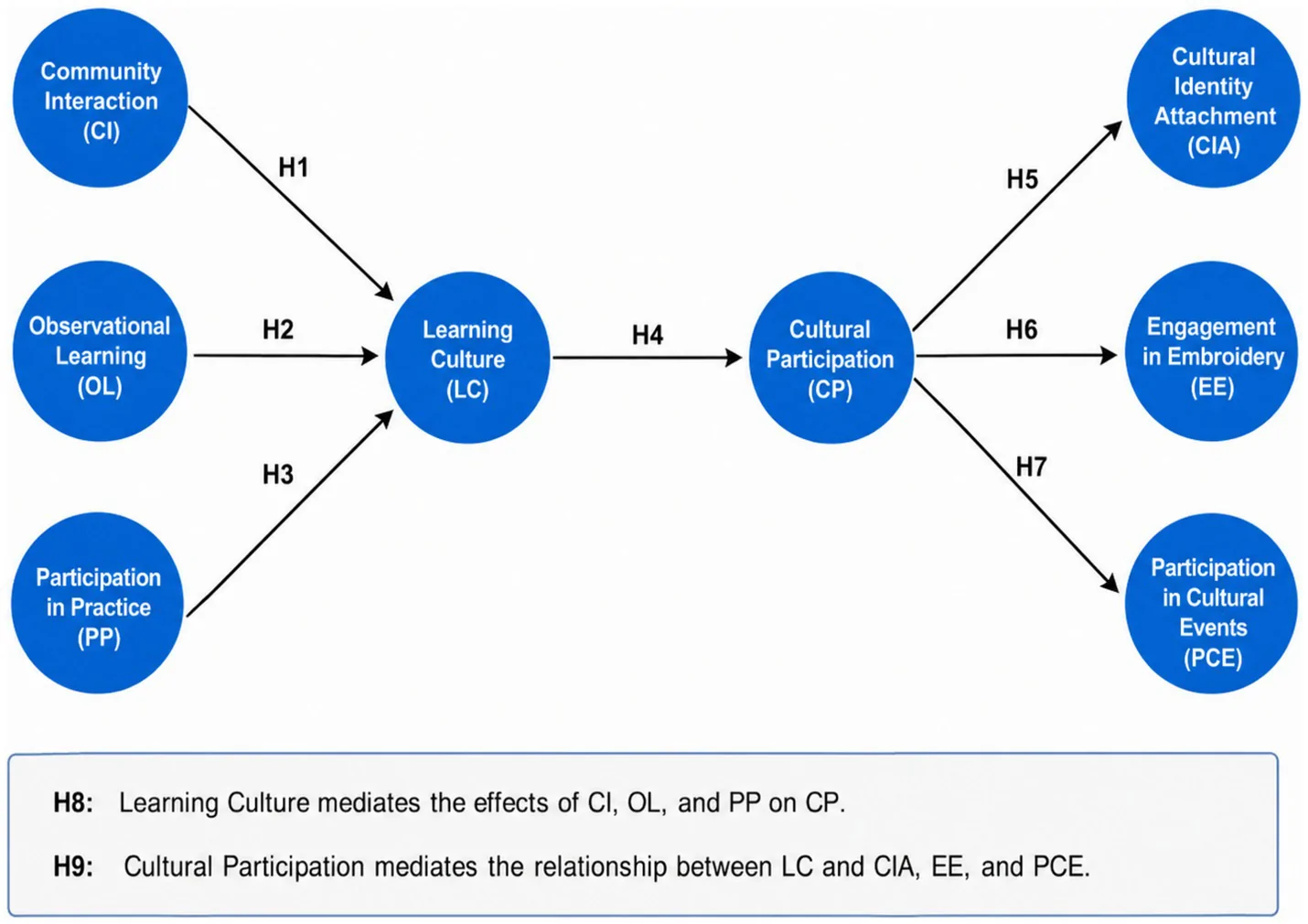
Conceptual model.

## Methods

In this research, an explanatory design was adopted to determine the causal relationships among social learning, cultural learning, and cultural Participation. In this research, a cross-sectional design was employed because the data were collected over a specific period and from respondents’ perspectives.

### Sampling methods and participants

This study used purposive sampling because the research required respondents with direct experience in Miao embroidery and Participation in community-based cultural activities. The sampling scope covered Miao embroidery communities in Guizhou Province, China, particularly those where embroidery is still practiced as part of household production, cultural transmission, and local heritage activities. Data collection was conducted during January–March 2026. The field investigation was conducted by approaching local embroidery communities, community coordinators, and active embroiderers familiar with embroidery production and cultural Participation.

Respondents were selected according to four inclusion criteria: (1) being at least 18 years old; (2) having at least 1 year of experience in Miao embroidery practice; (3) having participated in embroidery-related community or cultural activities; and (4) being willing to complete the questionnaire voluntarily. Before completing the questionnaire, respondents were informed about the purpose of the study, the voluntary nature of Participation, and the confidentiality of their responses. Questionnaires were distributed directly to eligible respondents during field visits and community-based embroidery activities. When necessary, brief explanations were provided to ensure that respondents understood the meaning of the questionnaire items, without influencing their answers.

A total of 280 valid responses were obtained and used for analysis (See Table 1). This sample size was considered adequate for PLS-SEM because it exceeded the minimum requirement per the 10-times rule and was sufficient for bootstrapping with 5,000 resamples. However, because the study used purposive sampling rather than probability sampling, the findings should be interpreted as representing the sampled Miao embroidery communities rather than all Miao embroiderers in China. This sampling strategy was appropriate for the study’s purpose, which focused on respondents with direct and relevant experience in embroidery learning, practice, and cultural Participation.

**Table 1:** Sample characteristics (*N* = 280).

Characteristics	Category	Frequency (*n*)	Percentage (%)
Gender	Female	162	57.8%
	Male	118	42.2%
Age	18–25 years	48	17.1%
	26–40 years	102	36.4%
	41–60 years	96	34.3%
	> 60 years	34	12.2%
Education background	Elementary/middle school	62	22.1%
	Senior high school	133	47.2%
	Diploma/bachelor’s degree	85	30.7%
Length of involvement in embroidery	1–2 years	46	16.4%
	3–5 years	94	33.6%
	> 5 years	140	50%

### Measurements

All constructs in this study were specified as first-order reflective latent variables and measured using a five-point Likert scale ranging from 1 = strongly disagree to 5 = strongly agree. The measurement instrument was developed by adapting key concepts from social learning theory (Bandura, 1977), communities of Practice (Lave and Wenger, 1991, 1998), cultural reproduction theory (Bourdieu, 1984), and social identity theory (Tajfel and Turner, 1979). In line with the revised research model, social learning was not treated as a separate higher-order construct. However, it was operationalized through three observable mechanisms: community interaction, observational learning, and Participation in practice. These three constructs represent the concrete ways in which artisans learn, exchange knowledge, and internalize craft practices within Miao embroidery communities.

Before the main survey, the measurement instrument underwent item adaptation and pretesting to improve content validity, clarity, and contextual relevance. Initial items were developed by adapting concepts from social learning theory, communities of practice, cultural reproduction theory, and social identity theory. They were then adjusted to the context of Miao embroidery communities. The items were reviewed to ensure that they reflected the intended constructs and were understandable to respondents with different educational backgrounds. Because the study was conducted in a local Chinese cultural context, the questionnaire wording was checked for semantic clarity and cultural appropriateness. A small-scale pretest was then conducted with respondents who had experience with Miao embroidery. Feedback from the pretest was used to identify ambiguous wording, overlapping meanings, and items that were difficult to understand. Minor revisions were made to improve clarity, reduce redundancy, and ensure that each item represented only one conceptual meaning. No constructs were removed after the pretest, but several items were reworded to better fit the local embroidery context. The final version of the questionnaire was then used for the main survey, and all measurement items were subsequently evaluated through the reflective measurement model assessment in PLS-SEM.

*Community Interaction (CI)* was measured as the intensity and quality of social exchange among members of the Miao embroidery community. The construct encompasses discussions of embroidery techniques, sharing difficulties and solutions, peer feedback, and interactions that strengthen understanding of the values of Miao embroidery. Four reflective indicators (CI1–CI4) were used to measure this construct.

The construct of *Observational Learning (OL)* is the means by which artisans learn embroidery techniques through observing other, more experienced persons within the community. The concept of observational learning includes modeling, imitation, internalizing of techniques, and understanding of the motifs and standards in practice through observation. OL was measured using four reflective indicators (OL1–OL4).

The concept of *Participation in Practice (PP)* refers to involvement in embroidery as a form of community learning. This construct involves Participation in embroidery, continued engagement, dedication, and contribution to the embroidery practice. Four reflective indicators were used (PP1–PP4).

*Learning Culture (LC)* was measured as the degree to which collective learning had become institutionalized within the community. This construct reflects shared learning routines, knowledge-sharing norms, informal support, intergenerational transmission, and the stability of community-based learning practices. Learning Culture was measured using five reflective indicators (LC1–LC5).

*Cultural Participation (CP)* refers to how people engage in cultural practices associated with Miao embroidery. Unlike Participation in Practice, CP refers to cultural Participation, commitment to culture, and willingness to keep participating in cultural practices collectively. There are four reflective measures of CP, namely CP1–CP4.

*Cultural Identity Attachment (CIA)* refers to affective commitment and psychological attachment to one’s Miao cultural identity as reflected in embroidery activities (Yuan et al., 2026). The construct comprises pride in Miao culture, personal attachment to embroidery, commitment to preserving cultural traditions, and recognition of embroidery’s role in shaping one’s identity. CIA was assessed through four reflective indicators (CIA1-CIA4).

*Engagement in Embroidery (EE)* refers to the behavioral and skill-related aspects of Participation in embroidery. EE reflects practice intensity, technical focus of embroidery, commitment to skills improvement, and active involvement in embroidery activities. Four reflective indicators (EE1-EE4) were used in measuring EE.

*Participation in Cultural Events (PCE)* refers to Participation in cultural events related to Miao embroidery, such as fairs, exhibitions, and traditional festivals. PCE was differentiated from cultural Participation by focusing on Participation in cultural events. The construct was measured through four reflective indicators (PCE1-PCE4).

All measurement items were evaluated through the reflective measurement model assessment in PLS-SEM. Indicator reliability was assessed using outer loadings, internal consistency was evaluated using Cronbach’s alpha, and convergent validity was assessed using Average Variance Extracted. Discriminant validity was examined using the Heterotrait-Monotrait Ratio. The results confirmed that all constructs met the recommended thresholds for reliability and validity.

### Data analysis technique

The data analysis in this study used the Partial Least Squares Structural Equation Modeling (PLS-SEM) approach, with SmartPLS software. The PLS-SEM method was chosen because it is suitable for predictive research models, involves multidimensional latent constructs, and tests mediation relationships simultaneously. Furthermore, PLS-SEM does not require strict assumptions of multivariate normality and is relatively robust to medium sample sizes, thus appropriate for the characteristics of this research data (Hair et al., 2019; Suryanti et al., 2019; Hair et al., 2022).

Because all variables were collected from the same respondents via a self-reported questionnaire at a single time point, common method bias was considered a potential concern. Several procedural steps were taken to reduce this risk. Respondents were informed that Participation was voluntary, their responses would remain confidential, and there were no right or wrong answers. The questionnaire was also organized into separate sections according to construct domains, and item wording was reviewed to reduce ambiguity and response consistency bias. In addition, common method variance was assessed statistically using Harman’s single-factor test and a collinearity-based assessment. Harman’s single-factor test indicated that no single factor explained the majority of the total variance. The full collinearity VIF values were also below the recommended threshold of 3.3, suggesting that common method bias was unlikely to be a serious threat to the interpretation of the results. Nevertheless, because the study relied on cross-sectional self-reported data, common method concerns cannot be completely ruled out and are acknowledged as a limitation.

The analysis procedure consisted of two main stages: measurement model assessment and structural model assessment. In the first stage, the reflective measurement model was evaluated through indicator reliability, convergent validity, internal consistency reliability, and discriminant validity. Indicator reliability was assessed using outer loadings, with values above 0.70 considered acceptable. Convergent validity was assessed using the Average Variance Extracted (AVE), with values above 0.50 indicating adequate convergence. Internal consistency reliability was evaluated using Cronbach’s Alpha, rho_A, and Composite Reliability, with values above 0.70 considered acceptable. Discriminant validity was assessed using the Heterotrait-Monotrait Ratio (HTMT), with values below 0.85 indicating satisfactory discriminant validity.

In the second stage, the structural model was tested to assess the strength and significance of the relationships among constructs. This evaluation was conducted by estimating path coefficients, the coefficient of determination (*R*^2^), and the effect size (*f*^2^). The significance of path coefficients and indirect effects was tested through a bootstrapping procedure with 5,000 resamples to obtain t-statistics and *p*-values. The mediation effects were examined using the indirect-effect approach. They were further interpreted by considering the relevant direct paths in an auxiliary mediation assessment to clarify the type of mediation.

## Result

### Structural model specification

Before assessing the measurement and structural results, the revised PLS-SEM model was specified based on the conceptual framework of this study. All latent variables were modeled as first-order reflective constructs, namely Community Interaction, Observational Learning, Participation in Practice, Learning Culture, Cultural Participation, Cultural Identity Attachment, Engagement in Embroidery, and Participation in Cultural Events. Social learning was not modeled as a separate higher-order construct; instead, it was operationalized through three observable mechanisms: Community Interaction, Observational Learning, and Participation in Practice.

The structural model tested seven direct hypotheses and two mediation hypotheses. The direct paths examined the associations between Community Interaction, Observational Learning, and Participation in Practice with Learning Culture; the association between Learning Culture and Cultural Participation; and the associations between Cultural Participation and three cultural outcomes: Cultural Identity Attachment, Engagement in Embroidery, and Participation in Cultural Events. Bootstrapping with 5,000 resamples was used to estimate path coefficients, t-statistics, *p*-values, and indirect effects.

Figure 2 presents the final PLS-SEM model. The model shows that Learning Culture is predicted by three social learning mechanisms, namely Community Interaction, Observational Learning, and Participation in Practice. Learning Culture is then linked to Cultural Participation, which is further associated with Cultural Identity Attachment, Engagement in Embroidery, and Participation in Cultural Events. This structure reflects the study’s main logic: social learning mechanisms are associated with cultural Participation through the formation of a learning culture. In contrast, cultural Participation serves as the proximal mechanism linking cultural outcomes in Miao embroidery communities.

**Figure 2:**
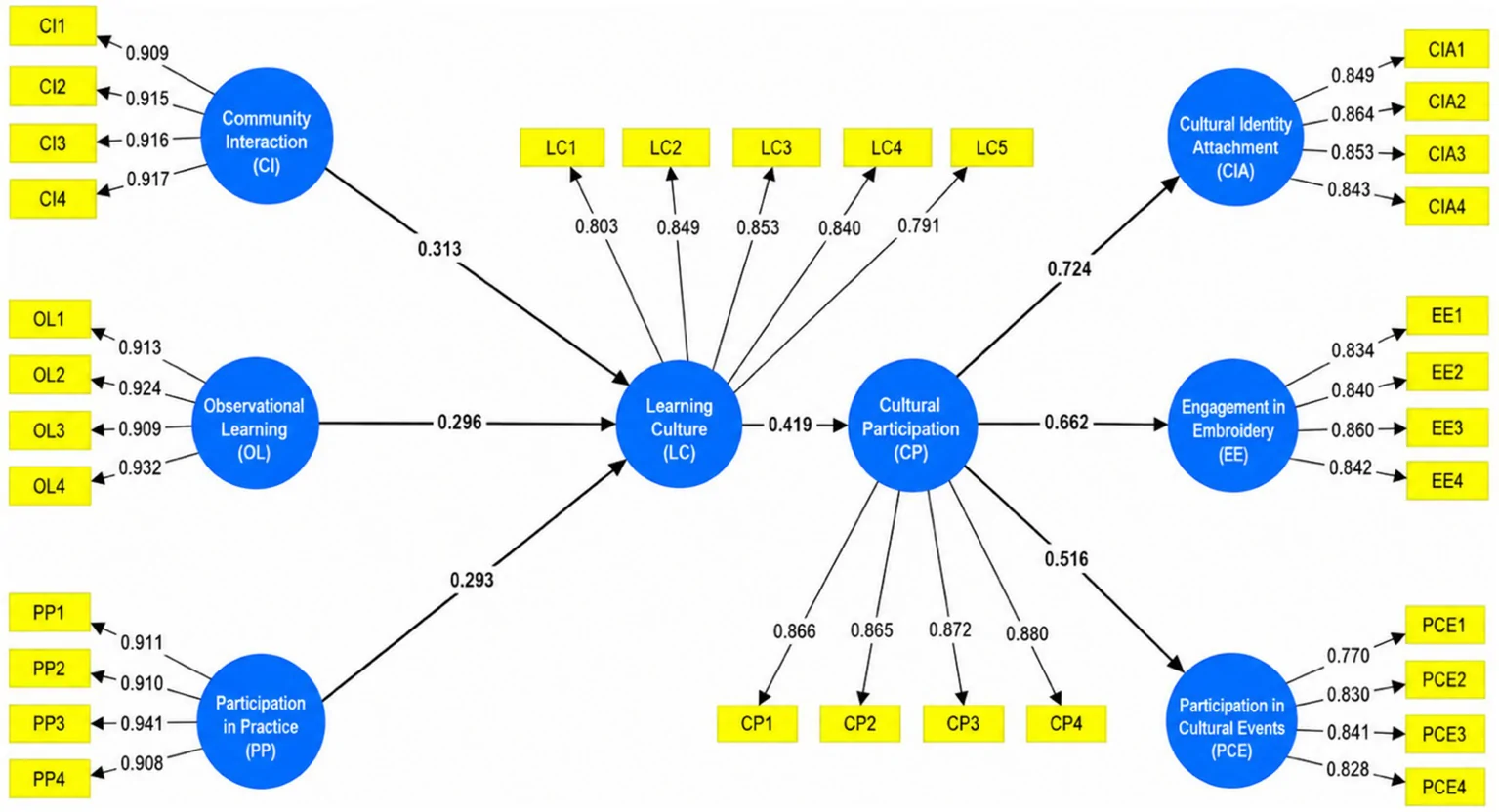
Final structural model using PLS-SEM. Figure 1 displays the standardized path coefficients (*β*) and *R*^2^ values for each endogenous construct. The values on the arrows indicate the strength of the relationship between the constructs, while the values in the circles indicate the proportion of variance explained by the predictor construct.

### Measurement model assessment

The measurement model was assessed to ensure that all reflective constructs met the criteria for indicator reliability, internal consistency, and convergent validity. In this revised model, eight first-order reflective constructs were evaluated: Community Interaction, Observational Learning, Participation in Practice, Learning Culture, Cultural Participation, Cultural Identity Attachment, Engagement in Embroidery, and Participation in Cultural Events. Social Learning was not assessed as a separate latent construct because it was operationalized through three mechanisms: Community Interaction, Observational Learning, and Participation in Practice.

The reliability of the indicators was determined using outer loadings. As shown in Table 2, all indicators had outer loadings exceeding the suggested criterion of 0.70. Indicators had a minimum loading of 0.770 and a maximum of 0.941. This shows that each indicator properly reflects the latent constructs. Therefore, there was no need to exclude any indicators from the model. The minimum loading, 0.770, was achieved by PCE1, and the maximum, 0.941, by PP3. Thus, the indicators used in this research have sufficient item reliability.

**Table 2:** Outer loadings.

Construct	Indicator	Outer loading
Community interaction (CI)	CI1	0.909
	CI2	0.915
	CI3	0.916
	CI4	0.917
Observational learning (OL)	OL1	0.913
	OL2	0.924
	OL3	0.909
	OL4	0.932
Participation in practice (PP)	PP1	0.911
	PP2	0.910
	PP3	0.941
	PP4	0.908
Learning culture (LC)	LC1	0.803
	LC2	0.849
	LC3	0.853
	LC4	0.840
	LC5	0.791
Cultural participation (CP)	CP1	0.866
	CP2	0.865
	CP3	0.872
	CP4	0.880
Cultural identity attachment (CIA)	CIA1	0.849
	CIA2	0.864
	CIA3	0.853
	CIA4	0.843
Engagement in embroidery (EE)	EE1	0.834
	EE2	0.840
	EE3	0.860
	EE4	0.842
Participation in cultural events (PCE)	PCE1	0.770
	PCE2	0.830
	PCE3	0.841
	PCE4	0.828

The reliability and convergent validity of the constructs were assessed via Cronbach’s alpha, composite reliability, and average variance extracted (AVE). From the results in Table 3, the Cronbach’s alpha scores range from 0.835 to 0.939, exceeding the 0.70 threshold. The same applies to the composite reliability scores, which range from 0.890 to 0.956. Therefore, high levels of internal consistency have been achieved.

**Table 3:** Construct reliability and convergent validity.

Construct	Cronbach’s alpha	Composite reliability (rho_a)	Composite reliability (rho_c)	AVE
Community interaction (CI)	0.934	0.934	0.953	0.836
Observational learning (OL)	0.939	0.946	0.956	0.846
Participation in practice (PP)	0.937	0.939	0.955	0.842
Learning culture (LC)	0.885	0.887	0.916	0.685
Cultural participation (CP)	0.894	0.894	0.926	0.758
Cultural identity attachment (CIA)	0.874	0.875	0.914	0.726
Engagement in embroidery (EE)	0.865	0.867	0.908	0.712
Participation in cultural events (PCE)	0.835	0.840	0.890	0.669

In terms of convergent validity, all constructs had AVE above the acceptable limit of 0.50, with their AVE scores falling between 0.669 and 0.846. Thus, each construct explained more than 50% of the variability in its indicators. Of all constructs, the highest AVE was observed for observational learning (0.846), while the lowest was for Participation in cultural events (PCE) (0.669). Notwithstanding having the lowest AVE score, PCE still had an acceptable score. Consequently, the model showed sufficient reliability and convergent validity.

### Explanatory power, effect size, and predictive relevance

The explanatory power of the structural model was assessed using the coefficient of determination (*R*^2^). As shown in Table 4, the model explained 32.2% of the variance in Learning Culture, indicating that Community Interaction, Observational Learning, and Participation in Practice jointly explained how learning culture is formed within Miao embroidery communities. The model explained 55.3% of the variance in Cultural Participation, indicating that Learning Culture accounted for a substantial proportion of the variation in Cultural Participation. Cultural Participation explained an additional 52.4% of the variance in Cultural Identity Attachment, 43.9% of the variance in Engagement in Embroidery, and 26.7% of the variance in Participation in Cultural Events. These results indicate that the model has moderate explanatory power for Learning Culture, Cultural Participation, Cultural Identity Attachment, and Engagement in Embroidery, and weaker but still meaningful explanatory power for Participation in Cultural Events.

**Table 4:** Coefficient of determination (R^2^) and predictive relevance (Q^2^).

Endogenous construct	*R* ^2^	Adjusted *R*^2^	*Q* ^2^	Interpretation
Learning culture (LC)	0.322	0.313	0.201	Moderate explanatory power
Cultural participation (CP)	0.553	0.549	0.361	Moderate to substantial explanatory power
Cultural identity attachment (CIA)	0.524	0.522	0.342	Moderate to substantial explanatory power
Engagement in embroidery (EE)	0.439	0.436	0.287	Moderate explanatory power
Participation in cultural events (PCE)	0.267	0.263	0.164	Weak to moderate explanatory power

Predictive relevance was assessed using *Q*^2^ values. A *Q*^2^ value above zero indicates that the model has predictive relevance for the corresponding endogenous construct. The *Q*^2^ results show that all endogenous constructs had *Q*^2^ values above zero, indicating that the model has acceptable predictive relevance for Learning Culture, Cultural Participation, Cultural Identity Attachment, Engagement in Embroidery, and Participation in Cultural Events.

The effect size (*f*^2^) was examined to assess the relative contribution of each predictor construct. As shown in Table 5, the effects of Community Interaction, Observational Learning, and Participation in Practice on Learning Culture were small to moderate, with *f*^2^ values of 0.141, 0.126, and 0.126, respectively. This suggests that Learning Culture is shaped by the combined contributions of multiple social learning mechanisms rather than by a single dominant predictor. Learning culture had a moderate effect on Cultural Participation (*f*^2^ = 0.215). The largest effects were found in the relationships between Cultural Participation and the three cultural outcomes, particularly Cultural Identity Attachment (*f*^2^ = 1.101), Engagement in Embroidery (*f*^2^ = 0.781), and Participation in Cultural Events (*f*^2^ = 0.364). These findings confirm that Cultural Participation is the most substantively important predictor in the model.

**Table 5:** Effect size (f^2^).

Structural path	*f* ^2^	Interpretation
Community interaction → Learning culture	0.141	Small to moderate
Observational learning → Learning culture	0.126	Small to moderate
Participation in practice → Learning culture	0.126	Small to moderate
Learning culture → Cultural participation	0.215	Moderate
Cultural participation → Cultural identity attachment	1.101	Large
Cultural participation → Engagement in embroidery	0.781	Large
Cultural participation → Participation in cultural events	0.364	Large

### Direct effects, hypothesis testing, and mediation analysis

Direct effect testing was conducted using a bootstrapping procedure with 5,000 resamples. The results show that all proposed direct relationships were positive and statistically significant. As presented in Table 6, Community Interaction had a positive effect on Learning Culture (*β* = 0.313, *t* = 5.542, *p* < 0.001), supporting H1. Observational learning also positively influenced Learning Culture (*β* = 0.296, *t* = 5.745, *p* < 0.001), supporting H2. Participation in practice also had a positive effect on Learning Culture (*β* = 0.293, *t* = 5.249, *p* < 0.001), supporting H3. These findings indicate that a single learning source does not shape the learning culture in Miao embroidery communities, but rather that it is the combined contributions of interaction, observation, and direct practice.

**Table 6:** Direct effects and hypothesis testing.

Hypothesis	Structural path	*β*	*t*-value	*p*-value	Decision
H1	Community interaction → Learning culture	0.313	5.542	<0.001	Supported
H2	Observational learning → Learning culture	0.296	5.745	<0.001	Supported
H3	participation in practice → Learning Culture	0.293	5.249	<0.001	Supported
H4	Learning culture → Cultural participation	0.419	7.205	<0.001	Supported
H5	Cultural participation → Cultural identity attachment	0.724	25.520	<0.001	Supported
H6	Cultural participation → Engagement in embroidery	0.662	18.415	<0.001	Supported
H7	Cultural participation → Participation in cultural events	0.516	10.104	<0.001	Supported

Learning culture had a positive and statistically significant effect on Cultural Participation (*β* = 0.419, *t* = 7.205, *p* < 0.001), which provided empirical evidence in favor of hypothesis four (H4). It implies that the process of cultural Participation will be positively influenced if there exists institutionalization of learning within communities. Furthermore, Cultural Participation had strong positive effects on all three cultural outcomes. It had the strongest effect on Cultural Identity Attachment (*β* = 0.724, *t* = 25.520, *p* < 0.001), followed by Engagement in Embroidery (*β* = 0.662, *t* = 18.415, *p* < 0.001), and Participation in Cultural Events (*β* = 0.516, *t* = 10.104, *p* < 0.001). Thus, H5, H6, and H7 were supported. These results demonstrate that cultural Participation is the most immediate and powerful predictor of cultural identity, practical engagement, and public cultural involvement.

Mediation analysis was conducted using bootstrapping with 5,000 resamples to examine the proposed indirect effects. As shown in Table 7, Learning Culture significantly mediated the relationships between the three social learning mechanisms and Cultural Participation. Community Interaction had a significant indirect association with Cultural Participation through Learning Culture (*β* = 0.131, *t* = 4.218, *p* < 0.001). Observational learning also showed a significant indirect association with Cultural Participation through Learning Culture (*β* = 0.124, *t* = 4.362, *p* < 0.001). Similarly, Participation in Practice had a significant indirect association with Cultural Participation through Learning Culture (*β* = 0.123, *t* = 4.166, *p* < 0.001). These findings support H8 and indicate that community interaction, observation, and practice are linked to cultural Participation through the institutionalization of learning into a shared learning culture.

**Table 7:** Mediation analysis.

Hypothesis	Indirect path	*β*	*t*-value	*p*-value	Decision
H8	Community interaction → learning culture → cultural participation	0.131	4.218	<0.001	Supported
H8	Observational learning → learning culture → cultural participation	0.124	4.362	<0.001	Supported
H8	Participation in practice → learning culture → cultural participation	0.123	4.166	<0.001	Supported
H9	Learning culture → cultural participation → cultural identity attachment	0.303	6.704	<0.001	Supported
H9	Learning culture → cultural participation → engagement in embroidery	0.277	6.451	<0.001	Supported
H9	Learning culture → cultural participation → participation in cultural events	0.216	5.979	<0.001	Supported

Cultural Participation also significantly mediated the relationships between Learning Culture and the three cultural outcomes. The strongest indirect association was found between Learning Culture and Cultural Identity Attachment through Cultural Participation (*β* = 0.303, *t* = 6.704, *p* < 0.001). The indirect association between Learning Culture and Engagement in Embroidery through Cultural Participation was also significant (*β* = 0.277, *t* = 6.451, *p* < 0.001), as was the indirect association between Learning Culture and Participation in Cultural Events through Cultural Participation (*β* = 0.216, *t* = 5.979, *p* < 0.001). These results support H9 and confirm that a learning culture is linked to cultural outcomes through active cultural Participation.

### Mediation type interpretation

To clarify whether the mediation effects were full or partial, an auxiliary mediation model was estimated by adding the corresponding direct paths from the social learning mechanisms to Cultural Participation and from Learning Culture to the three cultural outcomes. The results showed that the indirect effects remained significant after the inclusion of these direct paths. The mediation through Learning Culture was interpreted as partial mediation, because the social learning mechanisms still retained conceptual and empirical relevance in explaining Cultural Participation. However, their effects were transmitted primarily through Learning Culture. Similarly, the mediation through Cultural Participation was interpreted as partial mediation, because Learning Culture remained an important enabling structure. At the same time, Cultural Participation functioned as the behavioral mechanism linking Learning Culture to Cultural Identity Attachment, Engagement in Embroidery, and Participation in Cultural Events. Thus, the mediation results should not be understood as replacing the role of social learning mechanisms or learning culture, but as clarifying the sequential process through which learning becomes culturally consequential.

Overall, the mediation results support the model’s sequential logic. Community interaction, observational learning, and Participation in practice contribute to Cultural Participation through Learning Culture, which, in turn, links cultural outcomes to Cultural Participation. This finding confirms the central argument of the study: the sustainability of Miao embroidery is not associated with isolated learning activities alone, but with the transformation of social learning mechanisms into an institutionalized learning culture and their actualization through active cultural Participation.

## Discussion

From the findings, it can be stated that the culture of sustainability of Miao Embroidery communities is the result of a sequence of learning rather than learning alone. Community interaction, observational learning, and Participation in practice define the culture of learning, and the culture of learning, in turn, facilitates cultural Participation. The culture of Participation then emerges as the proximal factor influencing cultural identity, cultural Participation, and embroidery practices. From here, one can say that social learning processes will not guarantee cultural sustainability unless learning is institutionalized through a culture of learning, followed by a culture of Participation.

The first important finding concerns the role of social learning mechanisms in forming a learning culture. Community interaction, observational learning, and Participation in practice all had positive effects on learning culture, with relatively balanced coefficients. It indicates that the learning process in the Miao embroidery community is a combination of multiple learning practices rather than arising from a single leading mechanism. The interaction within the community allows for the exchange of knowledge among embroiderers, discussion of skills, feedback, and cultural interpretation. Observational learning facilitates the assimilation of models of right practice by observing expert practitioners. Engagement in practice ensures conversion of acquired knowledge into practice through repetition and informal learning. These findings contribute to the current literature on the role of tacit knowledge in crafts (Guo and Ahn, 2023; Chen and Wang, 2024) by identifying how the sustainable development of such skills requires not only knowledge transfer but also the transformation of interaction, observation, and practice into collective learning practices.

Learning culture’s effect on Participation in culture provides insights into the structural role played by learning in communities. Learning culture is not just a sign that learning practices are ongoing, but it is an indication that learning has been institutionalized as a norm within the community. With such norms of knowledge exchange and mentoring becoming prevalent, individuals would be more likely to view embroidery as a culturally significant practice. The significant effect of learning culture on cultural Participation, therefore, indicates that Participation is supported by a social environment that legitimizes, organizes, and sustains learning. This study advances debates on the role of social structures in cultural transmission (Chen and Wang, 2024; Mudau et al., 2024; He et al., 2024) by demonstrating that social structures serve an enabling role. Although learning the culture creates the prerequisites for engagement, cultural continuity will occur only if people act on these prerequisites.

The strongest finding of this study lies in the effects of cultural Participation on cultural outcomes. Cultural Participation had a particularly strong effect on attachment to cultural identity, followed by engagement in embroidery and Participation in cultural events. The conclusion that follows from the current result highlights that cultural identification not only arises from learning about culture but also involves Participation and repetition of culturally significant actions. About embroidery culture, Participation enables people to become acquainted with embroidery as a carrier of cultural memories, cultural symbols, and group identity. A strong connection between the variables under consideration suggests that levels of Participation and cultural commitment may help maintain the craft tradition. What is also noteworthy is that Participation in cultural activities affects involvement in embroidery, demonstrating people’s ability to transform everyday culture into cultural performance. The findings support previous studies, which have found that cultural Participation is associated with one’s identity and cultural connection (Grossi et al., 2019; Kim, 2020; Suryanti et al., 2021; Li et al., 2023). However, it provides an additional conclusion regarding how cultural Participation operates among craft community members – it is the means of maintaining the cultural link.

This finding also needs to be interpreted in relation to possible heterogeneity among embroiderers, particularly age and length of involvement in embroidery. Cultural Participation may not operate in the same way for all community members because craft learning and identity formation are shaped by generational position, accumulated experience, and access to different learning spaces. Older and more experienced embroiderers are likely to rely more strongly on embodied practice, family-based transmission, informal correction, and long-term community routines, which are central to the continuity of tacit craft knowledge (Aytekin and Rızvanoğlu, 2019; Guo and Ahn, 2023; Wang and Sun, 2022). Younger embroiderers, by contrast, may encounter Miao embroidery through more flexible and outward-facing channels, including training programs, cultural exhibitions, tourism-oriented activities, digital media, and market-related craft production (Du and Jiang, 2024; Zhang and Sharudin, 2024). These differences suggest that cultural Participation may function both as a mechanism of inherited continuity for experienced artisans and as a pathway of cultural identity formation and contemporary engagement for younger participants. Therefore, while the present model confirms the overall structural role of cultural Participation, future studies should examine age- and experience-based heterogeneity through multi-group analysis or comparative qualitative inquiry to determine whether the relationships among learning culture, cultural Participation, and cultural outcomes differ across generations and levels of embroidery experience.

The mediation results provide the study’s clearest theoretical contribution. Learning culture significantly mediated the relationships between the three social learning mechanisms and cultural Participation. This means that community interaction, observational learning, and Participation in practice contribute to cultural Participation only when they are organized into a stable learning culture. Learning culture mediates the relationship between learning culture and culture itself, suggesting that culturally learned learning culture is positively related to cultural outcomes through cultural Participation rather than normatively structured learning culture. Overall, the findings describe the order of mechanisms: Social learning mechanisms develop a learning culture; a learning culture enables Participation, which in turn leads to identification, crafting practices, and public participation. This finding goes beyond previous research, in which learning, Participation, and identity are discussed separately, and demonstrates how these concepts function as interrelated mechanisms of cultural heritage transmission.

Moreover, the current findings question the assumptions of modern heritage preservation, which overvalue digitization, design innovation, and the expansion of the market for traditional crafts (Zhang and Sharudin, 2024; Du and Jiang, 2024; Kofler and Walder, 2024). Though this approach could increase visibility, economic value, and public attention, it does not ensure the continuity of knowledge about traditional crafts. Without a learning culture and cultural Participation in society, the heritage of such knowledge can only be represented externally rather than socially reproduced. As a result, the current research emphasizes the need to prioritize sustainability over output-oriented preservation. Products, events, and digitized elements cannot ensure the sustainability of Miao embroidery. Instead, the mechanisms that contribute to the social reproduction of embroidery should be studied.

The current paper elaborates upon a microsociological explanation of cultural sustainability in craft villages. It is shown that the sustainability of Miao embroidery is contingent on interactions among learning mechanisms, learning culture, and cultural Participation. Learning mechanisms provide the necessary basis for the process, learning culture ensures its stability and organization, while cultural Participation turns it into reality. The introduction of learning culture as the mediating factor and of cultural Participation as the main driver of behavior results in a more specific approach to the reproduction of intangible cultural heritage.

## Limitations and future research

This study has several limitations that should be acknowledged. First, the use of a cross-sectional design limits the ability to examine temporal change and causal processes. Although the proposed model explains structural associations among social learning mechanisms, learning culture, cultural Participation, and cultural outcomes, it cannot determine whether these relationships develop sequentially over time. Future studies should use longitudinal designs to examine how learning culture and cultural Participation evolve across different stages of craft involvement and intergenerational transmission. Second, the study focused on Miao embroidery communities in Guizhou and used purposive sampling to reach respondents with direct embroidery experience. While this strategy was appropriate for the research objective, it limits the generalizability of the findings beyond the sampled communities. Future comparative studies should examine whether the proposed model applies to other Miao communities, other ethnic minority crafts, or different forms of intangible cultural heritage. Third, the data were collected via self-report questionnaires, which may introduce response bias and common method variance. Future research should combine survey data with interviews, participant observation, or ethnographic fieldwork to more deeply capture the lived processes of learning, correction, Participation, and identity formation. Fourth, this study did not conduct formal multi-group analysis by age, gender, educational background, or length of embroidery experience. Future studies should test whether the relationships among social learning mechanisms, learning culture, and cultural Participation differ across generations and levels of craft expertise. Such analysis would provide a more nuanced understanding of how traditional craft knowledge is reproduced, transformed, or weakened under changing social conditions.

## Conclusion

This study concludes that the sustainability of Miao embroidery is not associated with social learning mechanisms in isolation, but with a sequential process in which community interaction, observational learning, and Participation in practice are linked to collective learning culture, and learning culture is further associated with cultural Participation as a central mechanism of cultural continuity. The findings suggest that learning becomes culturally significant when it is embedded in shared norms, routines, and community practices, and when these learning structures are enacted through active Participation. Cultural Participation emerged as the strongest predictor of attachment to cultural identity, engagement in embroidery, and Participation in cultural events, indicating that intangible cultural heritage is sustained not only through preservation policies, digital visibility, or craft production but also through repeated and meaningful involvement in community-based cultural practice. By positioning learning culture as a mediating structure and cultural Participation as a key behavioral mechanism, this study offers a micro-sociological framework for understanding how traditional craft communities reproduce knowledge, identity, and cultural commitment under changing social conditions.

## Data Availability

The raw data supporting the conclusions of this article will be made available by the authors, without undue reservation.
